# The effect of a multimodal exercise program on paraspinal muscle morphology and clinical outcomes in chronic low back pain

**DOI:** 10.1177/09593020261419928

**Published:** 2026-03-19

**Authors:** Brent Rosenstein, Maxime Bergevin, Florian Bobeuf, Louis Bherer, Benjamin Pageaux, Mathieu Roy, Maryse Fortin

**Affiliations:** 1 Department of Health, Kinesiology and Applied Physiology, Concordia University, Montreal, Quebec, Canada; 2 École de kinésiologie et des sciences de l'activité physique (EKSAP), Faculté de médecine, Université de Montréal, Montreal, Quebec, Canada; 3 Centre de recherche de l'Institut universitaire de gériatrie de Montréal (CRIUGM), Montreal, Quebec, Canada; 4 Département de médecine, Université de Montréal, Montréal, Québec, Canada; 5 Centre de Recherche, Institut de Cardiologie de Montréal, Montreal, Quebec, CanadaCentre de Recherche, Institut de Cardiologie de Montréal, Montreal, Quebec, Canada; 6 Centre interdisciplinaire de recherche sur le cerveau et l’apprentissage (CIRCA), Montreal, Quebec, Canada; 7 School of Health, Concordia University, Montreal, Quebec, Canada; 8 Alan Edwards Centre for Research on Pain (AECRP), McGill University, Montreal, Quebec, Canada; 9 Department of Anesthesiology, McGill University, Montreal, Quebec, Canada

**Keywords:** low back pain, exercise therapy, paraspinal muscle, magnetic resonance imaging, function

## Abstract

**Background:**

Multimodal exercise programs do not specifically target lumbar paraspinal musculature, but incorporate aspects of different exercises to have an overall benefit.

**Objective:**

To investigate the effect of a multimodal exercise program on paraspinal muscle volume and composition, and patient outcomes in individuals with chronic low back pain (CLBP).

**Methods:**

Thirty-four participants with CLBP either completed a 14-week high-intensity training program (n = 8) including cardiorespiratory and resistance exercises 3 sessions per week or were waitlisted (n = 26). Participants underwent magnetic resonance imaging at baseline and post-intervention to assess paraspinal muscle volume (cm3) and fatty infiltration (% FI) at L3-L4, L4-L5, and L5-S1. Pain, disability, quality of life, pain-related fear (catastrophizing and Kinesiophobia), and anxiety were assessed via validated self-reported questionnaires.

**Results:**

Mixed-design ANOVA revealed no significant time × group interactions for paraspinal muscle volume and %FI. An exploratory analysis revealed a significant increase in multifidus %FI in the control group at L3-L4, L4-L5, and L5-S1, with a concomitant increase in multifidus volume at L3-L4 and L5-S1. The exercise group had a significant increase in multifidus %FI and volume at L5-S1. Significant time × group interactions for pain, disability, catastrophizing and kinesiophobia, and a main effect of group in physical and mental health were found. Significant correlations were found between changes in patient-reported and functional outcomes with paraspinal muscle morphology.

**Conclusion:**

Multimodal exercise programs may help prevent LBP-related paraspinal muscle atrophy and %FI, and lead to concomitant improvements in pain, disability and pain-related fear in individuals with CLBP.

## 1 Introduction

Low back pain (LBP) affected 619 million people globally in 2020, and is expected to reach 843 million by 2050.^1^ Recurrence is frequent as more than 60% of patients with an acute episode of LBP relapse within a year.^2^ Therefore, it is no surprise that it is the most common musculoskeletal condition worldwide.^3^ Out of 354 medical conditions, LBP is also the leading contributor to global productivity loss, work absenteeism, and years lived with disability.^3,4^ In addition, approximately 5–10% of LBP cases will develop chronic LBP (CLBP),^5^ which is defined as pain that persists for more than 3 months between the lower ribs and gluteal folds, with or without radiating leg pain.^6^ This form of LBP comes with significant healthcare-related and social costs.^7,8^ People with CLBP exhibit signs of muscular dysfunction and cardiorespiratory deconditioning, as evidenced by decreased strength,^9,10^ flexibility,^11,12^ endurance,^13^ and an increased association with obesity.^14^ Indeed, studies show a relationship between lumbar paraspinal muscle morphological changes (e.g., fatty infiltration, muscle atrophy, asymmetry) and LBP, especially in the multifidus muscle.^15–18^ Furthermore, there may be a relationship between paraspinal fatty infiltration (FI) and muscle volume, a 3D technique to measure muscle size. Interestingly, Snodgrass et al. found greater cervical multifidus volume and FI in chronic neck pain patients versus age and sex-matched asymptomatic controls.^19^ There was no difference between-groups in relative volume, a representation of volume without FI, which accounted for muscle, spinal level, side, sex, age and BMI. This proposes a similarity in lean muscle mass between people with chronic pain and pain-free people. Therefore, in accordance with previous studies, elevated amounts of FI in the multifidus muscle may be an LBP contributor.^18–21^ In addition, one study demonstrated that multifidus FI at the lower lumbar level may be associated with impaired self-reported and performance-based physical function in older patients with CLBP.^22^ Interestingly, no such relationship was found in pain-free individuals, which further emphasises the fact that a relationship between paraspinal muscle morphology and self-reported and functional outcomes may be unique feature of CLBP.^22^ Therefore, addressing paraspinal muscle morphological changes in individuals with CLBP could potentially lead to improved patient outcomes, such as pain, and function.

Exercise is commonly recommended as a first-line conventional treatment for CLBP, with systematic reviews supporting its effectiveness.^23,24^ More specifically, the literature supports the effectiveness of exercise in improving pain, pain-related fear, depression, function and quality of life in people with LBP.^25–29^ Among commonly used exercise interventions for LBP are motor control, trunk stabilization, and resistance training exercises,^30^ especially targeting lumbar paraspinal musculature. Literature regarding the benefits of different types of exercise on patient outcomes in CLBP is well documented. Recently, a network meta-analysis investigated the effects of different types of exercise on pain and function across 217 randomized controlled trials and 21,000 individuals with CLBP.^31^ The analysis revealed that Pilates, functional restoration training and McKenzie therapy were more effective than other types of exercise in improving pain and function. While the results imply these exercise types may have an edge in leading to improvements in patient outcomes, literature investigating the effects of exercise on improving paraspinal muscle morphology (e.g., hypertrophy, reversal of fatty infiltration) remains scarce and far less clear.

Multimodal exercise programs are comprehensive and incorporate aspects of different types of exercises to have an overall exercise benefit. Though this training program does not specifically target lumbar paraspinal musculature as in an isolated lumbar extension exercise program,^27^ it is still a comprehensive and functional exercise approach that solicitates the deep trunk muscles. Indeed, multimodal exercise has components of aerobic exercise, upper and lower body resistance training, abdominal strengthening, and stretching/flexibility. Consequently, each exercise component is beneficial for the rehabilitation of patients with CLBP by enhancing physical function.^25^ More specifically, aerobic exercise helps increase blood flow and nutrients to the spine, improving the healing process and decreasing LBP-related stiffness. Furthermore, resistance exercise and especially core strength training of deep abdominal muscles, which play a key role in supporting the lumbar spine, could improve spine stability and pain. Finally, stretching helps increase flexibility of tendons and ligaments in the back, leading to increased range of motion, mobilization and ultimately pain and function.^25^ Therefore, it possible that multimodal exercise program involving different aspects of training could be more effective in treating CLBP and benefit paraspinal muscle health. However, the literature in this area is limited. In addition, whether paraspinal muscle adaptations from exercise are associated with improved patient-reported and functional outcomes has yet to be determined.^32,33^

Altogether, the novelty of this study lies in its multimodal exercise approach, its integration of both physical and psychological dimensions, and its use of imaging to explore structural muscle changes following a multimodal exercise program. This study aimed to explore and advance the understanding of how multimodal rehabilitation may affect clinical outcomes and underlying muscle and psychological mechanisms in CLBP. Specifically, the aim of this study was to explore the effect of a 14-week multimodal exercise program versus a waitlist non-exercise control group on 1) paraspinal muscle size and composition (e.g., fatty infiltration), and 2) possible associations between changes in paraspinal muscle morphology with patient-reported and functional outcomes in individuals with CLBP. We hypothesized that multimodal exercise would prevent further LBP-related degeneration of paraspinal muscle size and composition. Furthermore, we hypothesized that the multimodal exercise intervention would lead to improvements in patient-reported and functional outcomes compared to the non-exercise control group. Finally, we expected that positive changes in muscle morphology in the exercise group would be associated with improvements in patient-reported and functional outcomes.

## 2 Methods

### 2.1 Study design and setting

This prospective observational study is a secondary analysis of a larger single-center prospective cohort study (Canadian Institutes of Health Research PJT-390119) that was conducted at the Centre de Recherche Institut Universitaire de Gériatrie de Montreal (CRIUGM). The study was approved by the Ethics Research Committee aging-neuroimaging research (# CER VN-16-17-11). All participants signed an informed consent form prior to beginning the study. After providing consent, participants were randomly allocated to a 14-week multimodal exercise program, or to a wait-list control group. Participants in the control group were offered to receive the intervention after the 14-week wait-time period. The effects of the exercise training program on pain and various psychosocial variables have been previously reported in studies with explicit different aims from the present study.^34,35^ These studies did not present the effects of the training program on paraspinal muscles, which has only been investigated in a population subsample.

### 2.2 Participants

Participants were recruited from advertisements on the website of the Quebec Association of Chronic Pain (“association québécoise de la douleur chronique” (AQDC)), or from the patient registry of the Quebec Low Back Pain Cohort.^36^ Participant inclusion criteria followed the Canadian minimum dataset for CLBP research.^37^ Included participants had CLBP (pain present for more than 3 months or pain for at least half of the days within the past 6 months) and a self-reported pain level of 4 or more over the past week. Excluded criteria included age below 18, following a multimodal exercise program for >30 min per week at the start of the study, history of diabetes, history of neurological or psychiatric disorders, presence of significant cardiorespiratory problems, and unstable signs of neuropathy (e.g., pain radiating in legs).

### 2.3 Sample size

Due to the COVID lockdown in Quebec, the sample size of the larger cohort study was limited to 57 participants. Out of 57 participants with CLBP enrolled in the larger study, 47 underwent lumbosacral MRI at baseline. The low number of lumbosacral MRIs is due to the paraspinal muscle sequence being added after the larger study began data collection. Overall, due to some participants not returning for the second scanning session (i.e., dropouts), 34 participants completed lumbosacral MRI at baseline and post-intervention, and were included in the current analysis.

### 2.4 Procedure

Participants completed two testing sessions on two separate days during the weeks immediately before and after the 14-week exercise intervention period. During the first testing session, participants completed a battery of functional tests to evaluate their physical and functional capacities. During the second testing session, participants completed a magnetic resonance imaging (MRI) scan. The first two weeks of the 14-week intervention were used to familiarize participants with the exercises. Both testing sessions were repeated after the 14-week period. Participants in the control group similarly came for the two testing sessions before and after the same 14-week period. Kinesiologists assisted training sessions one-on-one to ensure the safety of the participants. All exercise sessions took place at the CRIUGM gym facility.

### 2.5 Multimodal exercise intervention

The 14-week intervention involved 3 sessions per week of about 60 min, each separated by at least one day (Monday, Wednesday, Friday). Sessions 1–4 during the first 2 weeks were performed at a low-intensity, where the focus was on positioning, postural control, and exercise execution. The aim of this familiarization period was to avoid misrepresentative baselines or participant injury.

During sessions 5 and 6, maximal aerobic power (MAP) and one-repetition maximum (1RM) were determined to evaluate exercise capacity, and used to prescribe loads for sessions 7–42. As such, MAP was determined during an incremental test performed on a recumbent bike ergometer (Corival Recumbent, Lode B.V., Groningen, The Netherlands). Initial mechanical power was set at 50 watts for males and 35 watts for females. Power increased by 15 watts every minute, with a fixed pedaling cadence of 60 to 80 revolutions per minute, until volitional disengagement from the exercise. Maximal strength was assessed using the 1RM inertial method using three different machine-based exercises (Atlantic Inc. Laval, Quebec, Canada): leg press, seated chest press, and seated lateral pull down. The initial load was set at 90% of estimated 1RM, then increased by 2.5% to 5% after each successful repetition until failure.^38^

The exercise intervention included a warmup, resistance exercises and aerobic exercises. The start of the exercise intervention focused on strength endurance exercises, and the end focused on maximal strength. After the resistance component of each session, participants performed aerobic exercises, which involved high-intensity interval training exercises and incremental endurance training. After the 2-week low-intensity period in which lighter weights were used, the intensity for resistance exercises was gradually increased to 60% 1RM over the remaining 12-week period. Participants were encouraged to increase the load used for each exercise when they were able to do so without compromising the movement techniques. A kinesiologist progressively adjusted intensity throughout the intervention period to maximize physical improvements.

#### 2.5.1 Mondays and fridays

After the warm-up on Monday and Friday of each week, participants performed two circuits of resistance exercises. The first circuit focused on light strength-training, which consisted of 5 exercises: straight arm lateral pull down (15 repetitions), lateral shoulder raise (15 repetitions), an isometric exercise for abdominal muscles (30 s per side) using elastic bands, leg curl knee flexions (10 repetitions per leg) using a pulley, and a dorsal bridge hip extension exercise (15 repetitions) using body mass. The second circuit focused on heavy strength-training, which consisted of 4 machine-based exercises that alternated between the lower and upper body: leg press, leg curls on an exercise ball (leg extension and flexion), seated chest press and seated lateral pull down. The recovery period between exercises was the time needed to move from one station to another (approximately 30 s). Participants executed 3 circuit-sets of 15 repetitions with a resting time of 60 s between each circuit-set.

This resistance exercise component was followed by aerobic exercise, which involved high-intensity interval training exercise on a recumbent bike ergometer. This involved two sets of alternating between 60% MAP for 1 min and 80–85% MAP for 2 min. Participants then performed two 6-min sets of 15-s bouts of cycling at 100% MAP separated by active 15-s recovery periods at 60% MAP.

#### 2.5.2 Wednesdays

After the warm-up, participants performed 2 sets of the same 5 exercise light strength-training circuit as Monday and Friday. This resistance exercise component was again followed by aerobic exercises on a recumbent bike ergometer. This involved one set of a 25-min incremental protocol, which started at 65% MAP for the first 5 min, and was gradually increased 5% every 5 min until 85% MAP.

Each training session finished with stretching. Participants maintained the position of different stretches prescribed by the kinesiologist for 30–60 s depending on the time remaining in the session.

### 2.6 Data collection

All outcomes were acquired at baseline for both groups. All baseline assessments (e.g., MRI, questionnaires, functional outcomes) were repeated following the 14-weeks training or waitlist period. Self-reported questionnaires were completed on paper forms. Demographic characteristics were obtained at baseline.

### 2.7 Outcome measures

*Primary outcome measures:* 1) Multifidus muscle 3D volume and fatty infiltration (FI) at the L3-L4, L4-L5 and L5-S1 levels

*Secondary outcome measures:* 1) Erector spinae 3D volume and FI at the L3-L4, L4-L5 and L5-S1 levels, 2) pain, 3) disability, 4) quality of life, 6) kinesiophobia, 7) pain catastrophizing, 8) anxiety, and 9) a battery of functional tests including: trunk extension, spinal tenderness, 10-meter walk test, 6-min walk test, timed up-and-go (TUG), and lifting tasks.

### 2.8 Measurement tools

#### 2.8.1 MRI assessment of paraspinal muscle morphology

The MRI data was acquired on a Siemens Trio 3T scanner equipped with a 32-channel head coil. A DIXON sequence was used to acquire images of spinal discs from the L3 to S1 spinal levels. This technique provides quality images of paraspinal muscles allowing the analysis of size and composition. To calculate the collective 3D volume of each the left and right side, bilateral manual segmentation of regions of interest (ROI) signifying the multifidus and erector spinae cross-sectional area (CSA) were obtained on fat-water sequenced axial slices at L3-L4, L4-L5, and L5-S1. Three CSA measurements were obtained from three slices at the lower endplate of the top vertebra, the mid-disc, and the upper endplate of the bottom vertebra at each level and side. Each set of CSA measurements per level were used to compute the 3D volume. Thereafter, each right and left volume and %FI at each level were averaged to represent each level, and used in the analysis. Before the start of this study, the reliability of muscle volume and %FI for the multifidus and erector spinae were assessed from L1-L4 at each level. The rater (B.R.), a PhD student trained by a senior researcher (M.F.), randomly selected the images of 10 participants and independently segmented them. Following at least 5 days, the muscle segmentations were performed again. Excellent reliability was found, and is reported in a recent publication.^39^ Water and fat images were used to calculate the percent fat-signal fraction/FI using the equation: %FSF = (Signal_fat_/[Signal_water_ + Signal_Fat_] × 100) of each muscle at every level. Imaging analysis was performed using the Horos DICOM viewer software.

*Segmentation Method:* As demonstrated in another publication,^40^ the multifidus and erector spinae muscles were segmented separately in two ROIs. The posterior border of the multifidus was outlined along the epimysium, separate from the thoracolumbar fascia and adjacent adipose tissue. The medial border of the multifidus was outlined from the most superficial part of the spinous process to the deepest part where it adjoins the lamina, which included any fat within the area. The anterior deep border of the multifidus was outlined from the lateral lamina to the anterior mammillary process and zygapophyseal joint. These borders connected with the anterior deep borders of the erector spinae continuing to the lateral transverse process. A fatty streak, the intermuscular fascial border, between the multifidus (lateral border) and the erector spinae (medial border) outlined from the mamillary process to the posterior border represented the distinction between the two muscles. The fatty streak was included in the ROI of the erector spinae. The lateral border of the erector spinae was along the fascial border of the iliocostalis. The fascial plane outlined the posterior border of the erector spinae, which *included* present epimuscular fat tents between the longissimus and iliocostalis muscles. Epimuscular fat that was also present lateral to the iliocostalis muscle and below the lumbosacral fascia were also *included*.^41,42^ The 3D volume computation was conducted after the three independent CSA slice segmentations were completed per muscle and side at each level.

#### 2.8.2 Functional outcomes

Functional outcomes were evaluated on the first testing day prior to and after the intervention.

Functional tests included: 1) measure of total trunk extension, 2) evaluation of spinal tenderness, 3) 10-meter walk test, 4) 6-min walk test, 5) Timed Up-and-Go (TUG), and 6) lifting tasks.

##### 2.8.2.1 Trunk extension

For trunk extension, there were two trials each, in which an initial and final measure of total extension was taken between spinal levels C7 and S1 with a measuring tape to calculate length in centimeters. The average was taken for each pair of trials, and the difference between the average initial and final measures of total extension was calculated.

##### 2.8.2.2 Spinal tenderness

For spinal tenderness, gradually at each spinal level from T12 to S2, an evaluator pressed their thumb down on the spine of a participant lying prone and asked if there is pain. The participant either replied “yes” or “no”. The number of total “yes” and “no” replies were summed up, and a yes/no ratio was calculated.

##### 10-meter walk test

For the 10-meter walk test, there were three trials each, in which the usual and fastest gait time to walk 14 meters in a straight line was recorded in seconds. The distance to walk the first 10 meters was recorded with timing gates (TC-System, Browser Timing Systems, Draper, Utah, USA). The average of the final two trials was calculated for analyses. This test is crucial for the CLBP population as they have been shown to have slower gait speeds compared to healthy controls^43^ and normative data.^44^

##### 2.8.2.3 6-min walk test

For the 6-min walk test, the participant was instructed to walk the furthest they could back and forth down a 30-meter long hallway for 6 min. The total distance traveled was calculated in meters. This test has been validated and shown good reliability to assess function in individuals with diverse musculoskeletal disorders.^45,46^

##### 2.8.2.4 Timed up-and-go

For the timed up-and-go test, three trials were done to measure the usual gait time (seconds) it took to get up from a seated position on a chair with armrests, stand up, walk around a cone situated 3 meters in front of the chair, and then sit back down. The average time of the final two trials was calculated for analyses. This test has been validated and responsive in CLBP patients.^47,48^

##### 2.8.2.5 Lifting tasks

For the lifting task, participants were instructed to complete 18 different types of lifting tasks created from a combination of three positions and three weights of containers to lift off a table arranged in three rows.^49^ More specifically, standing in front of the table, participants could reach a container in the following positions: 1) for containers in the first row, with their body standing up straight and elbows in 90° flexion, 2) for containers in the second row, with their body upright but with their arms out straight by fully extending their elbow, and 3) for containers in the third row, with their arms out straight but slightly bending/forward leaning their body without touching the table or first containers. The weights were categorized as light/2.9 kg, medium/3.4 kg, and heavy/3.9 kg. For each task, the participant gave a pain rating on a scale from 0 (no pain) to 10 (excruciating/extreme pain). The sum of the pain ratings from all tasks was calculated.

#### 2.8.3 Questionnaires

Pain, disability, quality of life, pain-related fear (catastrophizing and Kinesiophobia), and anxiety were assessed by self-reported questionnaires. Participants completed the 36 Item Short-Form Health Survey questionnaire (SF-36), Oswestry Low Back Pain Disability Index (ODI) and numerical pain rating scale (NRS) to assess quality of life, disability and pain, respectively. The Pain Catastrophizing Scale (PCS) and the Tampa Scale of Kinesiophobia (TSK) were used to assess pain-related fear. The State Trait Anxiety Inventory (STAI) was used to measure anxiety. The questionnaires have demonstrated a suitable level of test-retest reliability and have been validated in CLBP.^37,50–57^

### 2.9 Statistical analysis

Means and standard deviations were calculated for baseline characteristics, and comparison of means between groups were assessed using an independent *t* test and chi-squared test for continuous and categorical variables, respectively. Means and standard errors were used to report all outcome measures. Mixed-design analysis of variance (ANOVA) were used to evaluate changes from pre- to post-intervention in paraspinal muscle size and composition, and self-reported patient outcomes. Time was used as a within-subject factor and group as a between-subjects factor. In all analyses, Levene's test confirmed that the assumption of homogeneity of variances was met. Nonparametric partial correlations evaluated the association between changes in paraspinal muscle morphology and changes in self-reported patient outcomes, and between changes in paraspinal muscle morphology and changes in functional outcomes. The combined lumbar levels (L3-L4, L4-L5, and L5-S1) were used to analyze the associations between muscle morphology and patient outcomes. More specifically, the right and left volumes and %FI for each muscle were averaged and summed at each level to represent the combined change in paraspinal muscle morphology. The strength of correlation coefficients (r) was categorized using Cohen's guidelines as 0.1 to 0.2, 0.3 to 0.5, and >0.5 interpreted as weak/small, moderate/medium and strong/large correlations, respectively.^58^ The IBM SPSS version 28.0 (IBM Corp., Armonk, NY, USA) was used to complete all statistical analyses; a *p*-value of <0.05 was considered statistically significant.

## 3 Results

### 3.1 Participants

Participant's baseline characteristics are presented in Table 1. A total of 34 participants (19 female, 15 male) with LBP consented to participate at baseline. All baseline characteristics were comparable between the exercise and control group except for the physical component of quality of life (SF-36 Physical). Physical quality of life was higher in the exercise group (*p* = 0.018).

**Table 1.** Participants’ baseline characteristics.

	Total (*n* = 34)	Exercise (*n* = 8)	Control (*n* = 26)	*p*-Value
Age (y)	44.0 ± 13.0	46.9 ± 17.0	43.2 ± 11.8	0.489^#^
Range	(21–73)	(25–73)	(21–70)	
Female, *n* (%)	19 (56)	5 (63)	14 (54)	0.666^†^
Height (cm)	169.5 ± 8.9	169.3 ± 10.7	169.6 ± 8.5	0.947^#^
Weight (kg)	79.1 ± 16.3	78.5 ± 18.7	79.4 ± 15.9	0.899^#^
BMI (kg/m^2^)	27.5 ± 4.6	27.2 ± 4.7	27.6 ± 4.6	0.834^#^
Range	(20.2–37.6)	(21.5–34.2)	(20.2–37.6)	
LBP Duration, *n*				0.383^†^
>5 years	19	6	13	
1–5 years	12	2	10	
<1 year	3	0	3	
LBP NRS (0–10)	4.8 ± 2.0	4.8 ± 1.7	4.8 ± 2.1	0.981^#^
ODI^a^	23.5 ± 10.9	19.4 ± 9.8	24.8 ± 11.1	0.262^#^
^*^SF-36 Physical^b^	59.3 ± 18.1	73.2 ± 13.5	55.5 ± 17.4	**0**.**018**^#^
SF-36 Mental^b^	72.0 ± 19.9	84.0 ± 11.3	68.8 ± 20.6	0.073^#^
PCS^a^	19.3 ± 11.7	16.1 ± 10.9	20.4 ± 12.0	0.416^#^
TSK^a^	26.8 ± 7.0	25.9 ± 8.1	27.1 ± 6.7	0.690^#^
STAI Trait^a^	39.2 ± 12.1	35.6 ± 12.9	40.3 ± 11.9	0.375^#^
STAI State^a^	34.9 ± 11.5	31.0 ± 10.0	36.1 ± 11.8	0.315^#^

Values are presented as means ± standard deviations, unless otherwise denoted. BMI: body mass index; LBP: lower back pain; NRS: Numerical Rating Scale; ODI: Oswestry Disability Index; PCS: Pain Catastrophizing Scale; STAI: State Trait Anxiety Inventory; SF-36: 36-item Short Form Health Survey; TSK: Tampa Scale of Kinesiophobia.

* – p < 0.05,

# Based on independent samples t-test.

† Based on chi-square test.

^a^ – 5 missing data point (1 from exercise, 4 from control).

^b^ – 1 missing data point (from exercise).

### 3.2 Effect of exercise and control on muscle volume

Table 2 shows changes in paraspinal muscle volume in the exercise and control group. A main effect of time was found in multifidus volume at L3-L4 (*p* = 0.031, $\eta_{p}^{2}$ = 0.146) and L5-S1 (*p* = 0.001, $\eta_{p}^{2}$ = 0.328). No time × group interaction was found for multifidus and erector spinae volume at L3-L4, L4-L5 or L5-S1 (all *p's* > 0.187, $\eta_{p}^{2}$ < 0.064). An exploratory analysis revealed a significant increase in multifidus volume in the control group at L3-L4 (0.33 [0.10 to 0.56] cm^3^) and at L5-S1(0.41 [0.05 to 0.77] cm^3^). Furthermore, the exercise group also had a significant increase in multifidus volume at L5-S1 (0.89 [0.25 to 1.53] cm^3^). No other significant changes in multifidus or erector spinae volume were observed for either group.

**Table 2.** Pre- to post-intervention changes in multifidus and erector spinae muscle volume.

	Exercise n = 8		Control n = 24				
	Pre	Post	Pre	Post	Main effect of time	Main effect of group	Time × group interaction
*L3-L4 Level*							
Multifidus volume (cm^3^)	6.64 (0.58)	6.83 (0.62)	7.28 (0.34)	7.61 (0.36)	*p* = 0.031 F = 5.11 df = 1 $\eta_{p}^{2}$ = 0.146	*p* = 0.307 F = 1.08 df = 1 $\eta_{p}^{2}$ = 0.035	*p* = 0.527 F = 0.409 df = 1 $\eta_{p}^{2}$ = 0.013
	MD (95% CI) 0.19 (−0.22, 0.59)		MD (95% CI) 0.33 (0.10, 0.56)^*^				
Erector spinae volume (cm^3^)	23.56 (1.80)	23.32 (1.79)	24.20 (1.04)	24.23 (1.03)	*p* = 0.646 F = 0.215 df = 1 $\eta_{p}^{2}$ = 0.007	*p* = 0.707 F = 0.144 df = 1 $\eta_{p}^{2}$ = 0.005	*p* = 0.547 F = 0.372 df = 1 $\eta_{p}^{2}$ = 0.012
	MD (95% CI) −0.24 (−1.02, 0.55)		MD (95% CI) 0.03 (−0.42, 0.49)				
*L4-L5 Level*							
Multifidus volume (cm^3^)	10.34 (0.73)	10.55 (0.72)	10.81 (0.42)	11.03 (0.41)	*p* = 0.116 F = 2.63 df = 1 $\eta_{p}^{2}$ = 0.080	*p* = 0.568 F = 0.334 df = 1 $\eta_{p}^{2}$ = 0.011	*p* = 0.964 F = 0.002 df = 1 $\eta_{p}^{2}$ = 0.000
	MD (95% CI) 0.21 (−0.27, 0.70)		MD (95% CI) 0.23 (−0.05, 0.50)				
Erector spinae volume (cm^3^)	19.64 (1.14)	19.82 (1.13)	19.65 (0.66)	19.67 (0.65)	*p* = 0.734 F = 0.117 df = 1 $\eta_{p}^{2}$ = 0.004	*p* = 0.959 F = 0.003 df = 1 $\eta_{p}^{2}$ = 0.000	*p* = 0.782 F = 0.078 df = 1 $\eta_{p}^{2}$ = 0.003
	MD (95% CI) 0.18 (−0.86, 1.22)		MD (95% CI) 0.02 (−0.58, 0.62)				
*L5-S1 Level*							
Multifidus volume (cm^3^)^a^	12.86 (0.83)	13.76 (0.97)	12.70 (0.47)	13.11 (0.55)	*p* = 0.001 F = 13.18 df = 1 $\eta_{p}^{2}$ = 0.328	*p* = 0.696 F = 0.156 df = 1 $\eta_{p}^{2}$ = 0.006	*p* = 0.187 F = 1.83 df = 1 $\eta_{p}^{2}$ = 0.064
	MD (95% CI) 0.89 (0.25, 1.53)^*^		MD (95% CI) 0.41 (0.05, 0.77)^*^				
Erector spinae volume (cm^3^)^a^	12.37 (1.22)	12.36 (1.23)	13.36 (0.69)	13.50 (0.70)	*p* = 0.842 F = 0.041 df = 1 $\eta_{p}^{2}$ = 0.002	*p* = 0.444 F = 0.603 df = 1 $\eta_{p}^{2}$ = 0.022	*p* = 0.820 F = 0.053 df = 1 $\eta_{p}^{2}$ = 0.002
	MD (95% CI) −0.01 (−1.13, 1.11)		MD (95% CI) 0.14 (−0.50, 0.77)				

CI: Confidence Interval; MD: Mean Difference.

* The mean difference is significant at the 0.05 level.

^a^ – 3 missing data point (1 from exercise, 2 from control).

### 3.3 Effect of exercise and control on fatty infiltration (% fat infiltration)

Table 3 shows changes in paraspinal muscle FI in the exercise and control group. A main effect of time was found in multifidus %FI at L3-L4 (*p* = 0.005, $\eta_{p}^{2}$ = 0.237) and L5-S1 (*p* = 0.007, $\eta_{p}^{2}$ = 0.242). No time × group interaction was found for multifidus and erector spinae %FI at L3-L4, L4-L5 or L5-S1 (all *p's* > 0.425, $\eta_{p}^{2}$ < 0.021). An exploratory analysis revealed a significant increase in multifidus %FI in the control group at L3-L4 (1.01 [0.26 to 1.77]), L4-L5 (1.30 [0.15 to 2.45]), and L5-S1 (1.38 [0.10 to 2.65]). Furthermore, the exercise group also had a significant increase in multifidus %FI at L5-S1 (2.34 [0.08 to 4.60]). For erector spinae %FI, the control group had a significant increase at L5-S1 (1.81 [0.03 to 3.59]). No other significant changes in multifidus or erector spinae %FI were observed for either group.

**Table 3.** Pre- to post-intervention changes in multifidus and erector spinae muscle % fatty infiltration.

	Exercise n = 8		Control n = 24				
	Pre	Post	Pre	Post	Main effect \hspace*{2.2pc}of time	Main effect of group	Time × group interaction
*L3-L4 Level*							
Multifidus %FI	23.96 (4.14)	25.19 (4.15)	27.35 (2.39)	28.37 (2.39)	*p* = 0.005 F = 9.30 df = 1 $\eta_{p}^{2}$ = 0.237	*p* = 0.496 F = 0.474 df = 1 $\eta_{p}^{2}$ = 0.016	*p* = 0.764 F = 0.091 df = 1 $\eta_{p}^{2}$ = 0.003
	MD (95% CI) 1.24 (−0.07, 2.54)		MD (95% CI) 1.01 (0.26, 1.77)^*^				
Erector Spinae %FI	40.05 (4.53)	40.48 (4.45)	39.75 (2.62)	40.12 (2.57)	*p* = 0.455 F = 0.572 df = 1 $\eta_{p}^{2}$ = 0.019	*p* = 0.949 F = 0.004 df = 1 $\eta_{p}^{2}$ = 0.000	*p* = 0.956 F = 0.003 df = 1 $\eta_{p}^{2}$ = 0.000
	MD (95% CI) 0.43 (−1.44, 2.29)		MD (95% CI) 0.37 (−0.71, 1.45)				
*L4-L5 Level*							
Multifidus %FI	28.58 (4.80)	28.97 (4.64)	32.64 (2.77)	33.94 (2.68)	*p* = 0.144 F = 2.25 df = 1 $\eta_{p}^{2}$ = 0.070	*p* = 0.412 F = 0.693 df = 1 $\eta_{p}^{2}$ = 0.023	*p* = 0.425 F = 0.655 df = 1 $\eta_{p}^{2}$ = 0.021
	MD (95% CI) 0.39 (−1.60, 2.38)		MD (95% CI) 1.30 (0.15, 2.45)^*^				
Erector spinae %FI	44.22 (4.67)	45.12 (4.53)	45.95 (2.70)	47.22 (2.61)	*p* = 0.106 F = 2.77 df = 1 $\eta_{p}^{2}$ = 0.085	*p* = 0.719 F = 0.132 df = 1 $\eta_{p}^{2}$ = 0.004	*p* = 0.782 F = 0.078 df = 1 $\eta_{p}^{2}$ = 0.003
	MD (95% CI) 0.91 (−1.41, 3.22)		MD (95% CI) 1.27 (−0.07, 2.61)				
*L5-S1 Level*							
Multifidus %FI^a^	30.99 (4.90)	33.33 (4.59)	33.82 (2.76)	35.19 (2.59)	*p* = 0.007 F = 8.63 df = 1 $\eta_{p}^{2}$ = 0.242	*p* = 0.668 F = 0.188 df = 1 $\eta_{p}^{2}$ = 0.007	*p* = 0.454 F = 0.578 df = 1 $\eta_{p}^{2}$ = 0.021
	MD (95% CI) 2.34 (0.08, 4.60)^*^		MD (95% CI) 1.38 (0.10, 2.65)^*^				
Erector spinae %FI^a^	48.73 (4.35)	50.40 (4.07)	49.93 (2.45)	51.74 (2.30)	*p* = 0.059 F = 3.88 df = 1 $\eta_{p}^{2}$ = 0.126	*p* = 0.791 F = 0.071 df = 1 $\eta_{p}^{2}$ = 0.003	*p* = 0.938 F = 0.006 df = 1 $\eta_{p}^{2}$ = 0.000
	MD (95% CI) 1.67 (−1.49, 4.83)		MD (95% CI) 1.81 (0.03, 3.59)^*^				

CI: Confidence Interval; FI: Fatty Infiltration; MD: Mean Difference.

* The mean difference is significant at the 0.05 level.

^a^ – 3 missing data point (1 from exercise, 2 from control).

### 3.4 Self-reported patient outcomes

#### 3.4.1 Pain, disability, and quality of life

Table 4 shows changes in pain, disability, and quality of life in the exercise and control group. A main effect of time was found in pain levels (*p* < 0.001, $\eta_{p}^{2}$ = 0.339) and disability (*p* = 0.011, $\eta_{p}^{2}$ = 0.239). A time × group interaction was found for pain (NRS) and disability (ODI) (all *p's* < 0.046, $\eta_{p}^{2}$ > 0.155). A significant improvement in self-reported pain levels (NRS) and in disability (ODI) was only found in the exercise group. For quality of life, there was a significant main effect of group in physical (SF-36 Physical) and mental (SF-36 Mental) health. There were no main effect of time (all *p's* > 0.100, $\eta_{p}^{2}$ < 0.091) or time × group interaction (all *p's* > 0.296, $\eta_{p}^{2}$ < 0.038). An exploratory analysis revealed a larger increase in physical and mental health in the exercise group.

**Table 4.** Pre- to post-intervention changes in pain, disability, and quality of life.

	Exercise n = 8		Control n = 24				
	Pre	Post	Pre	Post	Main effect of time	Main effect of group	Time × group
Pain (NRS)	4.75 (0.71)	2.50 (0.77)	4.77 (0.40)	4.62 (0.43)	*p* = <0.001 F = 16.42 df = 1 $\eta_{p}^{2}$ = 0.339	*p* = 0.189 F = 1.80 df = 1 $\eta_{p}^{2}$ = 0.053	*p* = 0.001 F = 12.49 df = 1 $\eta_{p}^{2}$ = 0.281
	MD (95% CI) −2.25 (−3.31, −1.19)^*^		MD (95% CI) −0.15 (−0.74, 0.43)				
Disability (ODI)^a^	20.00 (4.66)	11.00 (3.50)	24.70 (2.55)	23.50 (1.92)	*p* = 0.011 F = 7.54 df = 1 $\eta_{p}^{2}$ = 0.239	*p* = 0.058 F = 3.97 df = 1 $\eta_{p}^{2}$ = 0.142	*p* = 0.046 F = 4.41 df = 1 $\eta_{p}^{2}$ = 0.155
	MD (95% CI) −9.00 (−15.73, −2.27)^*^		MD (95% CI) −1.20 (−4.88, 2.48)				
SF-36 Physical^b^	73.24 (6.46)	84.85 (6.95)	55.06 (3.49)	57.74 (3.76)	*p* = 0.100 F = 2.90 df = 1 $\eta_{p}^{2}$ = 0.091	*p* = 0.001 F = 12.65 df = 1 $\eta_{p}^{2}$ = 0.304	*p* = 0.296 F = 1.13 df = 1 $\eta_{p}^{2}$ = 0.038
	MD (95% CI) 11.61 (−3.50, 26.72)		MD (95% CI) 2.67 (−5.49, 10.83)				
SF-36 Mental^b^	83.97 (7.23)	88.29 (7.36)	68.06 (3.90)	70.90 (3.97)	*p* = 0.361 F = 0.862 df = 1 $\eta_{p}^{2}$ = 0.029	*p* = 0.031 F = 5.15 df = 1 $\eta_{p}^{2}$ = 0.151	*p* = 0.849 F = 0.04 df = 1 $\eta_{p}^{2}$ = 0.001
	MD (95% CI) 4.32 (−9.55, 18.18)		MD (95% CI) 2.84 (−4.65, 10.32)				

CI: Confidence Interval; MD: Mean Difference; NRS: Numerical Rating Scale; ODI: Oswestry Disability Index; SF-36: 36-item Short Form Health Survey.

* The mean difference is significant at the 0.05 level.

^a^ – 8 missing data point (2 from exercise, 6 from control).

^b^ – 3 missing data point (1 from exercise, 2 from control).

#### 3.4.2 Psychosocial factors

**Figure 1.**
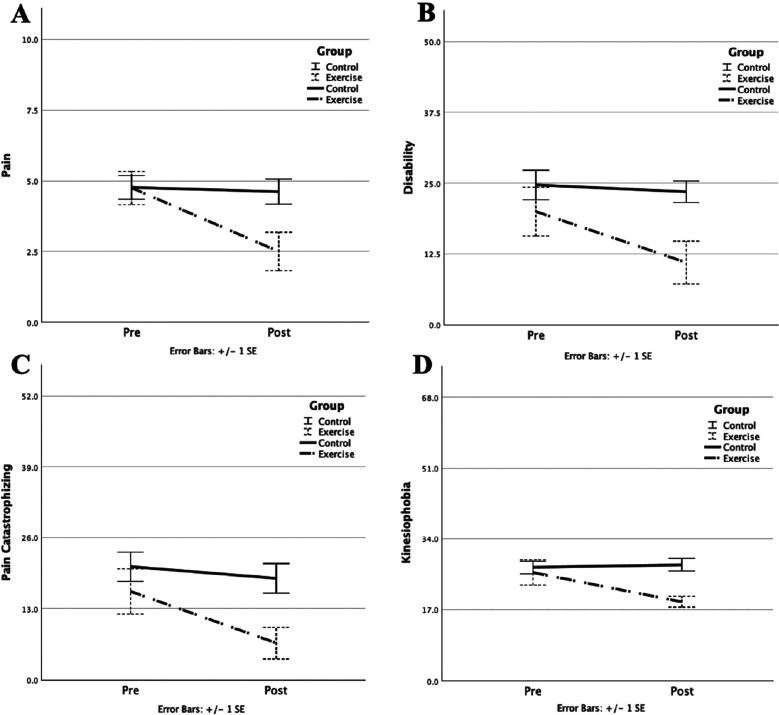
Changes in pain (A), disability (B), pain catastrophizing (C), and kinesiophobia (D) in the exercise (dashed line) and control (solid line) groups. Standard errors(error bars) are shown.

Table 5 shows changes in psychosocial factors (catastrophizing, kinesiophobia, and anxiety) in the exercise and control group. A main effect of time was found in catastrophizing (*p* = 0.001, $\eta_{p}^{2}$ = 0.334) and in kinesiophobia (*p* = 0.008, $\eta_{p}^{2}$ = 0.244). A time × group interaction for pain catastrophizing (PCS) and kinesiophobia (TSK) (all *p's* < 0.031, $\eta_{p}^{2}$ > 0.166) was found. A significant improvement in catastrophizing (PCS, *p* = 0.002) and in kinesiophobia (TSK) was only found in the exercise group. However, no significant results in anxiety (STAI) were found in either group. Figure 1 shows the changes in pain (A), disability (B), pain catastrophizing (C), and kinesiophobia (D) in the exercise and control groups.

**Table 5.** Pre- to post-intervention changes in psychosocial factors.

	Exercise n = 8		Control n = 24				
	Pre	Post	Pre	Post	Main effect of time	Main effect of group	Time × group interaction
Pain catastrophizing (PCS)	16.14 (4.51)	6.71 (4.34)	20.67 (2.60)	18.52 (2.51)	*p* = 0.001 F = 13.05 df = 1 $\eta_{p}^{2}$ = 0.334	*p* = 0.104 F = 2.83 df = 1 $\eta_{p}^{2}$ = 0.098	*p* = 0.031 F = 5.17 df = 1 $\eta_{p}^{2}$ = 0.166
	MD (95% CI) −9.43 (−15.13, −3.73)^*^		MD (95% CI) −2.14 (−5.44, 1.15)				
Kinesiophobia (TSK)	25.86 (2.72)	18.86 (2.35)	27.10 (1.57)	27.71 (1.35)	*p* = 0.008 F = 8.38 df = 1 $\eta_{p}^{2}$ = 0.244	*p* = 0.074 F = 3.45 df = 1 $\eta_{p}^{2}$ = 0.117	*p* = 0.002 F = 11.95 df = 1 $\eta_{p}^{2}$ = 0.315
	MD (95% CI) −7.00 (−10.92, −3.08)^*^		MD (95% CI) 0.62 (−1.65, 2.88)				
STAI trait	35.57 (4.62)	32.71 (4.83)	40.76 (2.66)	41.05 (2.79)	*p* = 0.330 F = 0.986 df = 1 $\eta_{p}^{2}$ = 0.037	*p* = 0.213 F = 1.63 df = 1 $\eta_{p}^{2}$ = 0.059	*p* = 0.236 F = 1.47 df = 1 $\eta_{p}^{2}$ = 0.054
	MD (95% CI) −2.86 (−7.47, 1.75)		MD (95% CI) 0.29 (−2.38, 2.95)				
STAI state	31.00 (4.40)	32.57 (4.87)	36.29 (2.54)	38.33 (2.81)	*p* = 0.258 F = 1.34 df = 1 $\eta_{p}^{2}$ = 0.049	*p* = 0.291 F = 1.16 df = 1 $\eta_{p}^{2}$ = 0.043	*p* = 0.880 F = 0.023 df = 1 $\eta_{p}^{2}$ = 0.001
	MD (95% CI) 1.57 (−4.00, 7.15)		MD (95% CI) 2.05 (−1.17, 5.27)				

CI: Confidence Interval; MD: Mean Difference; PCS: Pain Catastrophizing Scale; STAI: State Trait Anxiety Inventory; TSK: Tampa Scale of Kinesiophobia.

* The mean difference is significant at the 0.05 level.

### 3.5 Correlation between changes in muscle morphology and self-reported patient outcomes

Table 6 shows the correlations between changes in muscle volume and %FI from all levels combined, and changes in pain, disability, quality of life and psychosocial factors from baseline to 14 weeks in the exercise group. A strong positive correlation was present between changes in erector spinae volume (all levels combined) and kinesiophobia TSK score (r = 0.83, *p* = 0.042).

**Table 6.** Correlations between changes in the muscle morphology (L3-L4, L4-L5 and L5-S1 levels combined) and changes in pain, disability, quality of life, and psychosocial factors in the exercise group (n = 7).

	ΔMultifidus volume	ΔErector spinae Volume	Δ Multifidus %FI	Δ Erector spinae %FI
ΔPain (NRS)	0.00	0.20	0.42	0.18
ΔDisability (ODI)^b^	0.21	0.56	0.05	0.21
ΔSF36-Physical^a^	0.09	−0.37	0.26	0.31
ΔSF36-Mental^a^	−0.14	0.14	−0.60	−0.26
ΔCatastrophizing (PCS)^a^	0.66	0.49	−0.37	−0.20
ΔKinesiophobia (TSK)^a^	−0.03	0.83^*^	0.31	0.77
STAI Trait^a^	0.06	0.23	0.46	0.64
STAI State^a^	0.06	0.23	0.32	0.55

FI: Fatty Infiltration; NRS: Numerical Rating Scale; ODI: Oswestry Disability Index; PCS: Pain Catastrophizing Scale; STAI: State Trait Anxiety Inventory; SF-36: 36-item Short Form Health Survey; TSK: Tampa Scale of Kinesiophobia.

* Indicates p < 0.05,

^a^ – 1 missing data point,

^b^ – 2 missing data point.

### 3.6 Correlation between changes in muscle morphology and functional outcomes

Table 7 shows the correlations between changes in muscle volume and %FI from L3-L4, L4-L5 and L5-S1 levels combined with changes in functional outcomes from baseline to 14 weeks in the exercise group. A change in multifidus (r = 0.89, *p* = 0.007) and erector spinae %FI (r = 0.86, *p* = 0.014) were both positively correlated to changes in pain during lifting. Additionally, there was a positive correlation between change in spinal tenderness and multifidus %FI (r = 0.78, *p* = 0.038).

**Table 7.** Correlations between changes in the muscle morphology (all levels combined) and change in functional tests in the exercise group (n = 7).

	ΔMultifidus volume	ΔErector spinae volume	ΔMultifidus %FI	ΔErector spinae %FI
ΔTrunk Extension (cm)	0.54	0.38	0.11	0.25
ΔSpinal tenderness Pain	−0.66	0.11	0.78^*^	0.64
Δ10-meter Walk test usual gait (s)	0.07	0.18	−0.29	0.00
Δ10-meter Walk test fast gait (s)	0.54	0.29	−0.39	−0.21
ΔTUG usual gait (s)	0.11	−0.04	0.14	0.11
ΔLifting task pain	−0.54	0.29	0.89^*^	0.86^*^
Δ6-min Walk test (m)	0.75	0.25	−0.21	−0.32

FI: Fatty Infiltration; TUG: Timed Up-and-Go.

* Indicates p < 0.05.

## 4 Discussion

The multimodal exercise program had positive effects on many relevant patient outcomes. Improvements were found in pain, disability, quality of life, catastrophizing and kinesiophobia, with no changes found in the control group. Consistent with the literature, multiple systematic reviews show the overall effects of exercise in improving pain, function and quality of life^25,59^ in people with CLBP, especially when compared to no treatment.^24,28,60^ Furthermore, exercise programs that incorporate aerobic exercise, resistance exercise and flexibility, such as ours, have also been shown to improve pain and function in patients with CLBP.^25^ Our findings in psychosocial outcomes are also in line with recent systematic reviews, which show evidence that exercise improves kinesiophobia^61,62^ and fear-avoidance behaviors in people with CLBP.^63^ Interestingly, a systematic review and meta-analysis found that psychological factors, such as catastrophizing and pain-related fear, were negatively associated with pain thresholds in individuals with spinal pain.^64^ This stresses the importance of addressing pain-related psychosocial factors in patients with CLBP when planning a treatment, as a relationship between recovery and psychosocial factors is possible. Participating in exercise training may encourage people with LBP to confront their pain-related catastrophizing and fears of movement.^63^ More specifically, pain self-efficacy is one's confidence in their functional capability despite their pain condition.^65^ In fact, the main goal of exercise for patients with CLBP who display unhelpful pain beliefs should be to get them back to function despite their pain condition.^33^ For this reason, more general exercises or exposure therapies may be recommended as opposed to exercises that focus on how a patient loads their spine.^33^ It is possible that, through an exercise-related increase in self-efficacy, our exercise group formed a resilient attitude regarding their pain condition, and gained feelings of self-improvement and empowerment.^66^ Indeed, an improvement in self-efficacy could lead to improved patient outcomes, such as catastrophizing, kinesiophobia, disability, and quality of life through a healthier self-view of one's pain condition.^67^ While literature regarding the beneficial effects of exercise on patient outcomes in individuals with CLBP is broad, the effects of exercise, especially multimodal exercise programs, on paraspinal muscle morphology (e.g., hypertrophy, reversal of fatty infiltration) is understudied and far less clear.

Our results suggest that participation in the multimodal exercise program may have helped prevent further degeneration in paraspinal muscle size in individuals with CLBP. Notably, over the 14-week period the non-exercise control group showed an increase in multifidus %FI at each level from L3-S1 and in erector spinae %FI at L5-S1. Interestingly, a concomitant increase in multifidus volume at L3-L4 and L5-S1 was also found in the control group. Our findings suggest that there may be a relationship between paraspinal FI and muscle volume. In fact, Snodgrass et al. found greater cervical multifidus volume and FI in chronic neck pain patients versus age and sex-matched asymptomatic controls.^19^ Indeed, no difference between-groups in relative volume was found, which accounted for muscle, spinal level, side, sex, age and BMI, a representation of volume without FI. This proposes a similarity in lean muscle mass between people with chronic pain and pain-free people. While this may seem counterintuitive based on findings from a recent systematic review and biobank study showing smaller paraspinal muscle size in CLBP,^18,68^ most studies only look at CSA and not 3D volume, which likely provide a more precise estimate of overall muscle size. Therefore, in accordance with previous studies, elevated amounts of FI in the multifidus muscle are an LBP contributor.^18–21^ As the control group was not taking part in an exercise intervention, the observed increase in paraspinal FI is likely explained by a natural continuous deconditioning process. An exploratory analysis suggests that the exercise group showed an increase in multifidus %FI with a concomitant increase in multifidus volume, but only at L5-S1, a level largely implicated in muscle degeneration.^69–71^ We believe participants had an increase in %FI despite partaking in an exercise program for two reasons. First, a large amount of data shows that paraspinal muscle FI is clearly more prominent at the lower lumbar levels compared to the upper levels in people with and without LBP.^18,72^ For instance, a 15-year longitudinal demonstrated more paraspinal muscle atrophy and FI at L5-S1 compared to L3-L4.^70^ The lower lumbar levels are the levels most implicated in failure, since most bodyweight is endured at the L5-S1 level, inducing greater strain,^70^ spinal pathology incidence and degenerative changes.^69,71^ Due to greater levels of FI potentially being more resistant to changes in morphology,^73^ the high levels of fat at L5-S1 could partially clarify our findings.^18^ Secondly, a recent systematic review concluded that exercise may not reverse paraspinal muscle FI in CLBP, and that past interventions were perhaps too brief and used inadequate loads to lead to changes in muscle quality.^74^ Again, high levels of FI featured in LBP,^18,20,21^ might be more resilient to changes in morphology.^73^ Hence, reversing paraspinal muscle FI through exercise, especially at the lower lumbar levels, most probably requires more frequent and lengthier high-intensity exercise sessions. Effective compositional changes would likely also involve proper recruitment of targeted muscles through specific exercises, followed by high-intensity resistance training.^33^ For these reasons, we believe our intervention was not able to decrease FI, especially at L5-S1, as it was not lengthy or specific enough. However, the multimodal exercise program did appear to prevent further LBP-related increase in FI at the L3-L5 levels. It is important to mention that inconsistent findings in CLBP literature may also be partly explained by psychological heterogeneity in the CLBP population, and variations in methods to assess paraspinal muscle morphology (size and FI).^33,75^ Altogether, it is best to tailor exercise treatments to the differing needs of those with CLBP, which could lead to better treatment outcomes.^33,76^

Whether changes in paraspinal muscle morphology following exercise are associated with improved patient-reported benefits and functional outcomes has yet to be determined. Our analysis between muscle morphology and patient-reported outcomes in the exercise group revealed a significant strong correlation between change in kinesiophobia and erector spinae volume. This correlation analysis should be interpreted with caution, due to the small sample size (n = 6) and inability to report the 95% CIs. However, it is possible many other true correlations may not have come out at a group level due to heterogeneity between patients. Upon further analysis of the data, some participants had a decrease in erector spinae volume with a decrease in kinesiophobia, while participants with an increase in erector spinae volume had no change or minor changes in kinesiophobia. As mentioned, perhaps a change in muscle volume is associated with a change in muscle %FI, and therefore a decrease in volume is a potential indicator of a decrease in %FI and vice versa. Indeed, one study found a significant association between physical activity fear-avoidance beliefs and erector spinae intramuscular fat, which remained significantly moderate after correcting for covariates.^77^ It is possible that individuals with CLBP and high fears of movement may terminate exercise movements prematurely in fear of pain or injury, especially if the exercise targets spinal loading. Pain-related fear or avoidance behaviors during physical activity may lead to compositional changes from the disuse of muscles, such as an increase in %FI. This may explain why our correlation between changes in erector spinae %FI and changes in kinesiophobia nearly approached significance (*p* = 0.072). Altogether, there are mixed results in the limited studies that have explored the relationship between changes in muscle morphology and changes in patient outcomes. One systematic review reported no relationship between changes in multifidus morphology from motor control training and changes in disability in people with LBP.^78^ Another study found a correlation between improvements in disability, anxiety and depression in LBP patients that had improvements in multifidus and erector spinae muscle size and composition from resistance training.^79^ While systematic reviews show the positive effects of exercise on improving pain, function and quality of life in people with LBP,^24,25,28,59,60^ we cannot yet assume that exercise-induced changes in muscle morphology directly led to these improvements in patient outcomes.

Our findings between changes in muscle morphology and functional outcomes showed significant strong correlations between change in lifting pain and both multifidus and erector spinae %FI, and between change in spinal tenderness and multifidus %FI in the exercise group. This presents a relationship between changes in paraspinal muscle composition and functional changes. Again, this analysis should be interpreted with caution, due to the small sample size (n = 7) and inability to report the 95% CIs, but also with true significant correlations potentially missed. Upon further investigation of the data, some participants had a decrease in multifidus and erector spinae %FI with a decrease in pain during lifting, whereas participants with an increase in multifidus and erector spinae %FI had no improvement or smaller improvements in lifting pain. Our findings also suggested that participants exhibiting a decrease in multifidus %FI had a decrease in spinal tenderness, while participants with an increase in multifidus %FI had no improvement or smaller improvements in spinal tenderness. Indeed, there is evidence to suggest that a higher amount of paraspinal muscle FI is linked to higher LBP-related pain intensity and disability.^16,18,20,21,80–82^ While the literature investigating the relationship between changes in muscle morphology and functional outcomes is also limited, there are some interesting findings relating to this topic. Sions et al. state that multifidus intramuscular fat may help explain both self-reported and performance-based physical function in older patients with CLBP.^22^ As multifidus intramuscular fat increases at L5, physical function decreases in older adults with CLBP, while no such relationships were found in people without CLBP. This emphasises that a relationship between paraspinal muscle morphology and function may be unique feature of CLBP.^22^ Recently, a study found that participants with higher scores on a pain-related psychological measure (task-specific perceived harmfulness) and participants with CLBP (compared to pain-free participants) each spent more time to perform a lifting task at a significantly slower lumbar spine velocity.^83^ When analyzing the subgroups of participants with CLBP combined with higher perceived harmfulness scores, the differences were more pronounced.^83^ Our correlations between kinesiophobia and erector spinae morphology may relate to our findings between lifting pain and %FI. As mentioned, pain-related behaviors may lead to compositional changes from disuse of muscles, which has been shown to have positive correlations with LBP, FI, pain intensity and disability.^16,21,80–82^^,84^ Additionally, a reduction in lean muscle tissue (due to an increased amount of FI) could limit spinal function and muscle force production.^85^ Indeed, recent studies reported a significant association between paraspinal muscle composition and lumbar strength (both in flexion and extension).^86,87^ Overall, individualizing treatments by considering the heterogeneity of the CLBP population, could lead to improvements in patient outcomes, which then may help improve paraspinal muscle morphology and function.

## 5 Limitations

One of the main limitations of this study was the small sample size, especially for the exercise group (n = 8) compared to the control group (n = 26), limiting our analyses. Due to the small sample size, the exploratory analyses on non-significant interaction of the mixed-design ANOVA and correlation should be interpreted with caution. Future trials could consider using single-system designs to provide additional insights into individual response patterns and help refine interventions. Despite randomization and the participants’ baseline characteristics being comparable between groups, the physical component of quality of life was significantly higher in the exercise group. Additionally, due to the nature of the study, participants were not able to be blinded and knew which group they were randomized in.

## 6 Conclusions

This study's exploratory analysis provided preliminary evidence suggesting that multimodal exercise may help prevent LBP-related paraspinal muscle atrophy and %FI, and lead to concomitant improvements in pain, self-reported disability and pain-related fear in individuals with CLBP. Moreover, it provided limited support about the effect of exercise on lumbar paraspinal muscle morphology and its relationship with patient-reported and functional outcomes.
